# Exploring the relationship between nursing students’ knowledge and attitudes towards climate change and their psychological distress: a cross-national investigation

**DOI:** 10.1186/s12912-024-01927-8

**Published:** 2024-04-29

**Authors:** Ali D. Abousoliman, Ateya Megahed Ibrahim, Hasan Abualruz, Hussein M. Magdi, Donia Elsaid Fathi Zaghamir, Ahmed Alhowimel, Ahmed Hashem El-Monshed, Heba E. El-Gazar, Mohamed A. Zoromba

**Affiliations:** 1 College of Nursing, Prince Sattam Bin Abdulaziz University, Al-Kharj, Saudi Arabia; 2 Faculty of Nursing, Kafr Elsheikh University, Kafr Elsheikh, Egypt; 3 Faculty of Nursing, Port Said University, Port Said, Egypt; 4 Faculty of Nursing, Al-Zaytoonah University of Jordan, Amman, Jordan; 5 Faculty of Nursing, Beni-Suef University, Beni-Suef, Egypt; 6 Department of Health and Rehabilitation Sciences, College of Applied Medical Science, Prince Sattam Bin Abdulaziz University, Al-Kharj, Saudi Arabia; 7 Faculty of Nursing, Mansoura University, Mansoura, Egypt; 8 College of Health and Sport Sciences, University of Bahrain, Manama, Bahrain

**Keywords:** Nursing students, Knowledge, Attitudes, Psychological distress, Climate change

## Abstract

**Background:**

Climate change poses a worldwide challenge with anticipated exacerbation in the future, resulting in irreversible consequences. Nursing students may be vulnerable to experiencing psychological effects associated with climate change.

**Aim:**

The current study aimed to investigate the relationship between nursing students’ knowledge and attitudes toward climate change and their psychological distress.

**Method:**

This descriptive cross-sectional study recruited 377 nursing students from three universities located in Saudi Arabia, Jordan, and Egypt in the Middle East. Data collection was conducted using scales for assessing nursing students’ knowledge and attitudes towards climate change and their psychological distress. Correlations were assessed and multiple regression analysis was conducted to explore the predictors of students’ psychological distress.

**Results:**

The current study showed that knowledge regarding climate change significantly and positively correlated to the attitude toward climate change (*r* = 0.213), then again, the score of psychological distress significantly and negatively correlated to the score of students’ knowledge and attitude regarding climate change (*r* = − 0.182 and − 0.110 respectively). Regression analyses showed that academic achievement had the strongest positive impact on students’ psychological distress, while knowledge regarding climate change and attitude toward climate change had negative impacts (*β* = 0.381, *β*=-0.205, and *β*=-0.045 respectively). Moreover, knowledge and attitude regarding climate change were found to be significant predictors of students’ psychological distress, collectively accounting for 18.2% of the observed variance.

**Conclusions/Implication for future practice:**

The findings highlight the importance of incorporating climate change into nursing education programs. By enhancing nursing students’ knowledge and attitudes towards climate change, there is potential to reduce their psychological distress. This study underscores the need for curriculum reforms that integrate climate change topics, aiming to foster a well-informed and resilient future nursing workforce.

**Supplementary Information:**

The online version contains supplementary material available at 10.1186/s12912-024-01927-8.

## Introduction

### Climate change: a global challenge

Climate change is a significant global challenge with significant effects on health and well-being. It is an urgent and complex global problem that affects individuals and communities worldwide. It is a noteworthy hazard to public health, leading to increased morbidity and mortality rates, and psychological distress. Climate change impacts are expected to worsen in the coming decades, leading to irreversible consequences, especially for vulnerable populations. The healthcare sector, including nurses and nursing students, has a critical role in addressing health consequences of climate change [1]. Climate change is associated with a range of negative psychological outcomes, including anxiety, depression, and stress [2].

### Psychological distress and climate change

Nursing students, like other young people, may be predominantly liable to the psychological impacts of climate change, given their future orientation and the potential for climate change to impact their career paths. It is crucial to offer a holistic approach to assist nursing students who are distressed due to climate change concerns, surpassing mere awareness. Hence, it is noteworthy that addressing the psychological impact of climate change knowledge and attitude among nursing students necessitates a comprehensive approach beyond their awareness alone [3]. This includes how much their awareness affects their well-being to provide support for students who may be experiencing distress related to climate change issues. Young people around the world are becoming more and more distressed about climate change. According to a 10-nation survey, 84% of respondents between the ages of 16 and 25 said they were at least somewhat concerned about climate change, and 45% said that their concerns harmed their day-to-day activities [4]. As future healthcare providers, nursing students have a significant role to play in addressing the impact of climate change on health outcomes. However, it is essential to recognize that nursing students may experience psychological distress related to their awareness and attitudes toward climate change issues [5].

### Previous research findings

Several studies have suggested that environmental concerns can lead to psychological distress, particularly in healthcare professionals [6, 7]. Nursing students, in particular, may experience high levels of psychological distress due to their heavy workload, clinical experiences, and academic demands, which may be exacerbated by concerns about climate change [8, 9]. A study found that nursing students who had higher levels of anxiety and stress related to climate change were more likely to engage in pro-environmental behaviors [10]. This suggests that addressing climate change’s psychological effects among nursing students may not only improve their well-being but also promote sustainable healthcare practices [11].

A study by Anåker et al. (2021) conducted a qualitative study among nursing students in Sweden, students perceived the future of humanity as “gloomy”, with a significant effect to their perceptions of climate change’s impact on public health [12]. Similarly, Onieva-Zafra et al. (2020) utilized a descriptive correlational design to explore the relationship between climate change perceptions and psychological distress among 190 nursing students in Spain. The study measured stress and anxiety using the Perceived Stress Scale (PSS) and the State-Trait Anxiety Inventory (STAI), revealing that students with a higher perception of climate change as a health hazard reported greater levels of stress and anxiety [13]. A mixed method study by Ergin et al. (2021), involving 154 and 19 nursing students in Turkey, evaluated the effects of global warming, climate change, their effects on health and the roles and responsibilities of nurses [3]. Another study conducted in Australia found that a climate change and health course significantly increased the knowledge of nursing students regarding climate change’s effects on health [14]. Moreover, a survey conducted among nurses in China included a sample of 3866 found that environmental concerns were positively associated with psychological distress [15]. Another cross-sectional study conducted among nursing students in Italy found that concerns about the environment and climate change were significant predictors of psychological distress [16].

Knowledge and attitude about climate change may be enriched through the incorporation of climate change into nursing education programs which may also have positive impacts on nursing students’ psychological well-being [17]. The incorporation of environmental sustainability and climate change into nursing education programs has gained increasing attention in recent years. This is partly due to the recognition that healthcare is an imperative provider of gas emissions of greenhouse, therefore has an accountability to take action to decrease its effect on the environment [18]. Nursing students are key stakeholders in this effort, as they will play a critical role in promoting sustainable healthcare practices and addressing the health effects of climate change in students’ future practice [19].

It is important to note that incorporating climate change into nursing education programs [20]. The benefits of this extend beyond knowledge and attitude changes. A study conducted by Damery et al. (2021) among healthcare professionals in the United Kingdom found that sustainability education led to changes in waste management practices in healthcare settings [21], another study conducted in five countries, including seven different universities and schools of nursing, found that nursing students who received climate change education had higher levels of knowledge and positive attitudes toward climate change [22]. This suggests that incorporating climate change into nursing education programs may also have practical benefits in terms of promoting sustainable healthcare practices.

### Rationale for the current study

Accordingly, the psychological influence of climate change is an emerging area of research and has important implications for nursing education and practice. Climate change is predicted to have significant impacts on global health, including the spread of infectious diseases and an increased frequency of extreme weather events. Nurses who are educated about these impacts are better equipped to recognize and respond to these health risks and to educate patients and communities about prevention and mitigation strategies. Therefore, a well-developed understanding of the association between knowledge and attitudes concerning climate change among nursing students and their psychological distress is critical for developing effective strategies for addressing climate change in healthcare settings [1, 23]. While some studies have been conducted on nursing students’ attitudes and knowledge of climate change, there is a deficiency of studies examining the relationship between nursing students’ attitudes and knowledge towards climate change and their psychological distress, especially in the Middle East region. To address this gap, we conducted a multinational study involving nursing students from Egypt, Saudi Arabia, and Jordan.

## Aim and hypotheses

Our study aimed to investigate the relationship between nursing students’ knowledge and attitudes toward climate change and their psychological distress. To frame our hypotheses, we draw upon the concepts from environmental psychology and health education, particularly the Theory of Planned Behavior (TPB) and the Stress Reduction Theory (SRT). The TPB suggests that a person’s behavior is directly influenced by their intention to perform the behavior, which in turn is affected by their attitudes, subjective norms, and perceived behavioral control [24]. Applied to climate change, this theory supports the notion that higher levels of knowledge about climate change can foster more positive attitudes towards taking action against it, thereby potentially reducing the sense of helplessness and psychological distress associated with climate change impacts. Furthermore, the SRT posits that exposure to nature and understanding of environmental processes can lead to reduced stress levels. In the context of climate change education, this theory implies that increased knowledge about climate change, despite its distressing implications, can equip students with a better understanding of how to mitigate its effects and engage in pro-environmental behaviors, thus reducing anxiety and stress related to feelings of helplessness and doom [25]. Therefore, based on these theoretical frameworks and supporting empirical evidence, we hypothesize that nursing students with higher levels of knowledge and positive attitudes toward climate change would experience lower levels of psychological distress.

## Method

### Study design and setting

This study employed a cross-sectional study with a descriptive-analytical analysis across three universities in the Middle East: Prince Sattam Bin Abdulaziz University in Saudi Arabia (KSA), Al Zaytoonah University of Jordan, and Mansoura University in Egypt. We selected Prince Sattam Bin Abdulaziz University in KSA, Al Zaytoonah University of Jordan, and Mansoura University in Egypt for several strategic reasons. Firstly, these universities are representative of diverse geographic and socio-economic contexts within the Middle East, providing a broad spectrum of insights into the nursing students’ experiences and perceptions across different cultural settings. Saudi Arabia represents a high-income country with significant investments in healthcare education, while Jordan and Egypt, as middle-income countries, present different educational and environmental challenges and opportunities. Moreover, the inclusion of these countries offers a unique perspective on climate change in the Middle East, a region particularly vulnerable to its impacts yet underrepresented in global climate change research. By focusing on these specific universities and countries, the study aims to fill a critical gap in the literature. The research followed the guidelines for reporting observational studies, as outlined in the Strengthening the Reporting of Observational Studies in Epidemiology (STROBE) Statement.

### Participants and sampling

This study was conducted on undergraduate nursing students from the five full-time levels. Data were collected during the second semester of the academic calendar 2023. The average expected completion time of the survey consumed 15–20 min. International students and internship students were excluded. A two-phase sampling strategy was employed to enrol staff. Phase 1, stratified sampling technique is employed during data collection to guarantee that the sample accurately reflects the diversity in the number of students at various universities. In Phase 2, convenience sampling was used to recruit students in each institution.

OpenEpi, Version 3, an open source calculator was used to determine the required sample size, by giving the total population size of nursing students in studied universities = 6596 during the first semester of 2023 (187, 832, and 5577 attending in Prince Sattam Bin Abdulaziz University, Al Zaytoonah University, and Mansoura University, respectively), using the formula Sample size *n* = [DEFF*Np(1-*p*)]/ [(*d*2/*Z*21-/2*(*N*-1) + p*(1-*p*)], the minimum sample size needed was 260, with the hypothesized% frequency of outcome factor in the population (*p*) = 50%+/-5, confidence limits as% of 100 (absolute +/-%)(*d*) = 10%, and design effect (for cluster surveys-*DEFF*) = 1. The assumption of a 50% frequency was employed as a conservative approach, the study guided by the principle of maximizing the sample size to accommodate the uncertainty associated with the prevalence of knowledge and attitudes towards climate change among nursing students. This frequency ensures the maximum sample size required for adequate statistical power. The required sample size from each university (strata) was; 11, 33, and 219 from Prince Sattam Bin Abdulaziz University, Al Zaytoonah University, and Mansoura University respectively. To make up for a projected dropout rate of or incomplete responses from our experience, we, therefore, made the most conservative assumption of a response rate of 50% [26]. The final sample size utilized for analysis was 377 students, achieving a response rate of 96%.

### Instruments of data collection

The questionnaire consisted of four sections. The first section assessed students’ demographics including gender, age, academic level, and academic grade suggesting achievement. The second section assessed students’ knowledge reading climate change phenomena using the Climate Change Knowledge Test (CCKT) developed by Gazzaz and Aldeseet, (2021) [27]. CCKT comprised three sub-sections that encompassed three sub-constructs or sub-scales, specifically addressing the understanding of (I) the nature, (II) causes, and (III) effects of climate change. Each of these sections encompassed ten true-false questions, where students’ true answers indicate better knowledge. Cronbach alpha reliability shed an acceptable degree at 0.75. The third section assessed students’ attitudes reading climate change phenomena using the Attitude of Research Scholars Towards Climate Change scale developed by Netravathia and Chauhan, (2014), the scale consisted of 12 statements (8 positive and 4 negative) as students’ responses were measured along a 5-point Likert scale ranging from 1 (Fully disagree) to 5 (Fully agree). The original co-efficient reliability of the scale showed 0.813, which represents acceptable reliability [28].

The translation and adaptation process of the CCKT and the Attitude of Research Scholars towards Climate Change scale was meticulously designed to ensure their linguistic and conceptual integrity while making them culturally relevant to Arabic-speaking participants. The last author, proficient in both English and Arabic, conducted the initial translation. This step ensured a preliminary translation faithful to the original while considering linguistic nuances. The criteria for selecting the bilingual translator for the back-translation included fluency in both languages, familiarity with the academic discipline’s terminology, and experience in cross-cultural research methodologies. The chosen translator met these criteria, bringing a depth of understanding to the translation process that facilitated the accurate conveyance of complex concepts.

Following back-translation, the versions were compared, identifying discrepancies that ranged from minor linguistic differences to more substantial conceptual misalignments. These discrepancies were addressed through iterative revisions, ensuring a high degree of equivalence with the original scales. The panel of expert nursing professors, selected based on their dual expertise in nursing education and bilingual proficiency, provided a critical review of the translations. Criteria for their selection included academic qualifications (Ph.D. in Nursing), publications in English, and experience in cross-cultural research. Their feedback led to specific modifications, such as the adaptation of certain terms to better reflect local cultural contexts and the clarification of items that could be misinterpreted. For example, the term ‘climate change’ was adapted to a culturally specific equivalent that encompasses local environmental concerns, enhancing the relevance and comprehensibility of the scales.

The pilot study conducted with 15 students not only tested the reliability of the translations but also served as a final check for clarity and cultural appropriateness. Feedback from this pilot group led to further refinements, such as simplifying complex terms and rephrasing questions for better comprehension. The reliability of the instruments, as confirmed by Cronbach’s alpha, and the confirmatory factor analysis (CFA) results, underscored the success of our translation and adaptation process in maintaining the integrity and applicability of the instruments. The reliability of the CCKT was assessed using Cronbach’s alpha, resulting in a value of 0.828. Similarly, the Attitude of Research Scholars towards Climate Change scale demonstrated a high level of internal consistency with a Cronbach’s alpha of 0.814 according to the pilot study, the results of the CFA in this study showed satisfactory ft: χ2 = 108.54, df = 54, χ2/df = 2.01, IFI = 0.94, TLI = 0.92, CFI = 0.93, RMSEA = 0.067.

The fourth section assessed psychological distress using Kessler Psychological Distress Scale (K10) developed by Kessler et al., (2002) [29]. The K10 is a self-report questionnaire consisting of 10 items rated on a 5-point scale (score range: 10–50) and is commonly used to assess levels of psychological distress. Respondents select the response that best reflects their experiences over the past four weeks. Higher scores on the K10 indicate higher levels of psychological distress. The reliability of the K10 was found to be high, with a Cronbach’s alpha coefficient of 0.93 [30].. As well a study found that the Arabic version of the K10 questionnaire, which has been used in the current study, also Cronbach’s alpha coefficient demonstrated a high internal consistency of 0.88 [31, 32].

### Data collection and data analysis

Data was collected using a convenience sampling method, by interviewing students in the identified settings. Students were given information about the study’s goal and who should take part, and then a printed copy of the questionnaires was distributed to the students who agreed to participate voluntarily in the study, uncompleted questionnaires were excluded. The final sample size that participated in the study was 377. Data were collected from January 2023 to June 2023.

To ensure the highest standards of data quality and minimize potential biases, several measures were implemented throughout the data collection process. Firstly, all interviewers involved in administering the questionnaires were thoroughly trained on the study’s objectives, the importance of maintaining neutrality, and the techniques for ensuring that responses were gathered without leading or influencing participants. This training included simulated interviews to standardize data collection procedures and ensure consistency across all interviewers. Secondly, to further standardize the data collection process and reduce variability, a detailed protocol was developed. This protocol outlined the step-by-step procedures for administering the questionnaires, including instructions on how to approach participants, secure informed consent, and ensure the completeness and confidentiality of the responses. Interviewers were equipped with identical sets of materials and followed the same script to introduce the study and its objectives to participants, thereby minimizing the risk of information bias.

Confidentiality and anonymity of responses were paramount. Participants were informed that their responses would be completely anonymous, with no identifying information collected. To reinforce this, each questionnaire was coded with a unique identifier, and the data were stored in a secure, password-protected database accessible only to the research team. These measures were designed to encourage honest participation by assuring students that their responses could not be traced back to them, thus reducing potential response bias. The combination of interviewer training, standardized data collection protocols, and strict confidentiality measures was integral to maintaining the integrity of the data collection process to ensure the reliability and validity of the data collected, providing a robust foundation for our analysis and findings.

Ethical consideration.

The students who were accessible and freely agreed to participate in the study had signed a consent form. The informed consent process involved providing participants with a concise overview and purpose of the study, assuring them of the confidentiality and anonymity of their data, and explicitly stating that the results would be exclusively utilized for scientific purposes. The authors obtained ethical approval to conduct the study from The Standing Committee of Bioethics Research (SCBR), at Prince Sattam bin Abdulaziz University, Approval No. (SCBR-094-2023) before ongoing collecting the responses from sample via questionnaires.

### Data analysis

Data analysis was performed using SPSS software version 26.0. Initially, the normality of the data was assessed using the one-sample Kolmogorov-Smirnov test, the data showed normal distribution, therefore parametric statistics were utilized. Categorical variables were summarized using frequencies and percentages, while continuous variables were presented as mean ± SD (standard deviation). For comparisons between variables, the Student t-test was employed. In addition, for more than two groups comparison the Analysis of Variance (ANOVA) test was used. Correlations between continuous were assessed using the Pearson correlation test. The multiple regression analysis was designed to identify predictors of students’ psychological distress, incorporating variables that showed relation with psychological distress such as academic achievement, knowledge regarding climate change, and attitudes toward climate change. Prior to conducting the multiple regression analysis, several preliminary analyses were performed to ensure that the assumptions underlying regression analysis were met. Firstly, the assumption of normality of residuals was verified by visual inspection of Q-Q plots, which confirmed that the residuals were approximately normally distributed across the range of predicted values. Secondly, to assess multicollinearity among the predictor variables, we calculated the Variance Inflation Factor (VIF) and Tolerance values for each variable. VIF values were all below 10, and Tolerance values were above 0.2, indicating that multicollinearity was not a concern for our regression model. Statistical significance was considered at *p* < 0.05, with smaller p-values indicating more significant results.

## Results

### Characteristics of the sample

Table 1 showed that the study comprised 213 female students (56.5%) and 164 male students (43.5%). Regarding age distribution, 215 participants (57%) were 20 years or younger, while 162 participants (43%) were older than 20 years. Academic achievements varied among students with 92 (24.4%) achieving grade C, 156 (41.4%) achieving grade B, and 129 (34.2%) achieving grade A. Studied students who were aged more than 20 years were significantly higher in their knowledge and their attitude toward climate change, also they were lower in their psychological distress rather than other students who were aged 20 years or less. Attitudes toward climate change of studied male students were significantly higher than females, while there was no significant difference according to gender in knowledge about climate change nor psychological distress experienced by studied students. Students who were studying in the third academic year were significantly lower in their knowledge about climate change and higher in their psychological distress than their peers. Moreover, students who achieved B academic achievement were higher in knowledge and attitude toward climate change, whereas, students who achieved C academic achievement were lower in their psychological distress than their colleagues.

**Table 1:** Variance of knowledge and attitude regarding climate change and psychological distress according to studied students’ sociodemographic and education characteristics (*n* = 377)

Sociodemographic And Education Characteristics	Total	Knowledge		Attitude		Psychological Distress	
		Mean (SD)	Test of Sig.	Mean (SD)	Test of Sig.	Mean (SD)	Test of Sig.
Overall	377						
Age (years):							
≤20	215	18.76 (2.9)	3.269^***^	40.70 (8.3)	2.610^**^	26.48 (9.4)	3.687^***^
>20	162	19.77 (3)		42.70 (6.6)		22.83 (9.6)	
Gender:							
Male	164	19.22 (2.9)	0.132	43.39 (6.6)	4.285^***^	24.19 (10.4)	1.285
Female	213	19.18 (4)		40.15 (8.1)		25.47 (9)	
Academic Year:							
Second	189	19.59 (3.2)	4.963^**^	41.97 (8.6)	0.570	23.12 (7.9)	16.392^***^
Third	90	18.40 (2.5)		41.18 (8.2)		29.76 (11.5)	
Fourth	98	19.16 (2.9)		41.10 (4.6)		23.93 (9.3)	
Academic achievement:							
C	92	18.15 (2)	13.432^**^	41.42 (7.4)	5.089^**^	18.93 (5.6)	31.065^***^
B	156	20.05 (2.8)	^*^	42.90 (6.9)		25.51 (11.6)	
A	129	18.91 (3.5)		40.03 (8.4)		28.47 (7.1)	

** & *** significant at ≤ 0.01 & ≤0.001; respectively

Table 2 presented that there was no significant difference according to the country of studied students’ age, knowledge about effects of climate change, total knowledge about climate change, and, Total score of psychological distress. Knowledge regarding the nature of climate change among Jordanian students was significantly lower than among other studied students, then again, knowledge regarding the causes of climate change among Egyptian students was significantly lower than among other studied students. Attitudes toward climate change of studied Egyptian students were significantly lower than Saudi or Jordanian students, on the other hand, there was no significant difference according to the country of studied students in knowledge about climate change nor psychological distress.

**Table 2:** Variance of studied variables of studied students according to their country (*n* = 377)

Studied variables	Location	n	Mean (SD)	Median	Range (Min-Max)	F
Age	KSA	59	20.71 (1.73)	20.00	9 (18–27)	1.271
	Jordan	91	20.41 (1.27)	20.00	4 (18–22)	
	Egypt	227	20.41 (1.27)	20.00	4 (18–22)	
	Total	377	20.45 (1.36	20.00	9 (18–27)	
Knowledge concerning the nature of climate change	KSA	59	6.05 (1.59)	6.00	5 (4–9)	20.850^***^
	Jordan	91	5.74 (1.47)	5.00	7 (3–10)	
	Egypt	227	6.87 (1.51)	7.00	5 (4–9)	
	Total	377	6.47 (1.59	6.00	7 (3–10)	
Knowledge concerning causes of climate change	KSA	59	7.00 (1.39)	7.00	6 (4–10)	21.828^***^
	Jordan	91	6.76 (1.45)	7.00	6 (4–10)	
	Egypt	227	5.73 (1.76)	6.00	7 (2–9)	
	Total	377	6.18 (1.72	6.00	8 (2–10)	
Knowledge concerning the effects of climate change	KSA	59	6.76 (1.44)	7.00	6 (4–10)	1.424
	Jordan	91	6.67 (1.31)	7.00	7 (3–10)	
	Egypt	227	6.45 (1.52)	6.00	6 (3–9)	
	Total	377	6.55 (1.46	6.00	7 (3–10)	
Total score of knowledge regarding climate change	KSA	59	19.81 (2.78)	20.00	14 (15–29)	1.545
	Jordan	91	19.16 (2.61)	19.00	16 (14–30)	
	Egypt	227	19.05 (3.17)	19.00	13 (13–26)	
	Total	377	19.20 (2.99	19.00	17 (13–30)	
Total score of Attitude regarding climate change	KSA	59	44.88 (9.13)	43.00	68 (24–92)	17.345^***^
	Jordan	91	43.87 (6.59)	44.00	36 (23–59)	
	Egypt	227	39.77 (7.09)	37.00	38 (22–60)	
	Total	377	41.56 (7.65	41.00	70 (22–92)	
Total score of Psychological distress	KSA	59	24.81 (9.97)	24.00	39 (10–49)	0.311
	Jordan	91	24.26 (9.65)	22.00	39 (10–49)	
	Egypt	227	25.20 (9.57)	23.00	39 (10–49)	
	Total	377	24.92 (9.63	23.00	39 (10–49)	

KSA = Kingdom of Saudi Arabia, F = ANOVA test, *** significant level at ≤ 0.001

In analyzing the correlations between variables as presented in Table 3, the negative correlation between age and academic achievement (-0.238**) could reflect a variety of factors, including, potentially, the different life responsibilities that older students might juggle alongside their studies. The significant positive correlation between knowledge about climate change and attitude towards it (0.213**) underscores the importance of incorporating comprehensive climate change education into nursing curricula, as understanding may foster a more proactive stance towards mitigation and adaptation strategies in healthcare settings. Conversely, the significant negative correlation between psychological distress and both knowledge and attitude towards climate change (-0.182** for knowledge and − 0.110* for attitude). This could indicate that empowerment through education may help mitigate some of the psychological impacts of climate change concerns among nursing students. Unexpectedly, we observed a positive correlation between academic achievement and psychological distress (0.366**), indicating that higher academic performance is associated with greater levels of distress. Possibly reflecting the high pressures and stress associated with academic excellence in competitive environments.

**Table 3:** Correlations between studied variables (*n* = 377)

Studied variables	1	2	3	4	5
Age	1				
Academic achievement	-0.238^**^	1			
Total score of knowledge regarding climate change	0.065	0.087	1		
Total score of attitude toward climate change	0.085	-0.056	0.213^**^	1	
Total score of psychological distress	0.032	0.366^**^	-0.182^**^	-0.110^*^	1

The results of the multiple regression analysis exposed in Table 4 indicated that academic achievement, knowledge regarding climate change, and attitude toward climate change were significant predictors of students’ psychological distress. Collectively, these predictors accounted for 18.2% of the total variance observed in students’ psychological distress. The statistical test used to assess the overall significance of the regression model was an F-test, with a resulting value of 27.598 and a significance level of *p* < 0.001, indicating that the model as a whole was highly significant.

The positive beta coefficient for academic achievement (β = 0.381) is particularly noteworthy, suggesting a significant positive relationship with psychological distress. This indicates that as students’ academic achievements increase, so does their level of psychological distress, highlighting the potential stressors associated with academic performance demands. The magnitude of this coefficient, being the highest among the predictors, underscores academic achievement as a critical factor in students’ psychological well-being. Conversely, the negative beta coefficients for knowledge regarding climate change (β=-0.205) and attitude toward climate change (β=-0.045) suggest that increased knowledge and more positive attitudes towards climate change are associated with lower levels of psychological distress. The stronger effect of climate change knowledge on distress reduction, as indicated by its larger absolute beta value compared to attitude, points to the importance of education and awareness in mitigating psychological distress related to climate change. These findings suggest that enhancing knowledge about climate change could serve as a protective factor against psychological distress among nursing students. Furthermore, the relatively smaller magnitude of the beta coefficient for attitude towards climate change (β=-0.045) compared to knowledge indicates that while positive attitudes towards climate change are beneficial, knowledge has a more substantial impact on reducing psychological distress. This distinction underscores the importance of educational interventions that not only foster positive attitudes but also increase knowledge about climate change to effectively support students’ mental health.

**Table 4:** Results of a multiple linear regression analysis of predictors of psychological distress of the studied students (*n* = 377)

Predicators	B	SE(B)	β	t	P value	95.0% Confidence Interval for B (lower / upper)	
Constant	-3.001	6.270	-	− 0.479	0.632	-15.33 / 9.328	
Academic achievement	0.510	0.063	0.381	8.078	< 0.001	0.386 / 0.634	
Knowledge regarding climate change	− 0.662	0.155	− 0.205	-4.263	< 0.001	− 0.967 / − 0.357	
Attitude toward climate change	− 0.057	0.061	− 0.045	− 0.940	0.348	− 0.176 / 0.062	

*R* = 0.426, R^2^ = 0.182; ΔR^2^ = 0.175; F = 27.598^***^; SE standard error, β standardized regression coefficient,

## Discussion

Climate change represents a paramount global challenge of our time. Its detrimental effects possess the capacity to yield catastrophic outcomes, endangering the very existence of humanity. Therefore, it is imperative for individuals, particularly those within the scientific community, to possess a comprehensive understanding of this issue and its potential remedies. By doing so, they can initiate vital transformations in economic systems, resource utilization, human behavior, and overall perspectives toward nature (30). The urgency of addressing the climate crisis and its impact on human health calls for prompt and adaptive measures from societies. Extensive research has examined climate change’ effects on health as findings underscore the importance of swift action and version to mitigate the climate change health consequences [6]. Emerging evidence supports the profound influence of climate change on the health of humans, through a particular focus on mental well-being [32, 33].

Numerous studies have established a correlation between climate change and the prevalence of significant psychiatric conditions. Gradual increases in average temperatures and aerosol concentrations (i.e., smoke, dust, and pollen) correlate with higher incidences of aggressive and violent behaviors, suicides, and psychiatric disorders [34–36]. Overall, an increase in psychiatric disorders and suicide rates is reported to be associated with all climate change-induced health risks. Extreme weather events like heatwaves, floods, or wildfires result in higher incidences of traumatic stress, general anxiety, depression, phobias, alcohol abuse, and drug impairment, as indicated by Gebhardt et al. (2023) [37]. Additionally, an expanding body of research indicates that the escalating temperatures resulting from climate change may significantly and detrimentally affect mental health. This is substantiated by a series of studies conducted by reputable researchers [38–43], demonstrating a robust connection between rising temperatures and an increased risk of suicidal behavior. These findings emphasize the pressing need for extensive exploration and comprehension of the psychological repercussions of climate change.

To our knowledge, our study is the first to investigate the relationships between climate change and mental health in nursing students. However, more studies shed light on this important topic in medical students in general [44, 45]. Their investigation examines the psychological effects of climate change awareness among medical students. A total of 203 medical students in Germany were surveyed regarding their awareness of the implications of climate change, and their mental health was assessed using established questionnaires for depression, anxiety, post-traumatic stress, and perceived stress. The findings suggest that while medical students experience significant perceived stress related to climate change, this stress does not yet manifest in depressive, anxious, or traumatic symptoms. However, climate-related perceived stress correlates negatively with potential resilience factors such as attachment style, structural abilities, and sense of coherence.

The absence of significant differences across countries in the studied variables opens up intriguing avenues for exploration and invites the author to consider various factors contributing to this remarkable similarity. One plausible explanation could be the presence of shared global influences that transcend geographical boundaries. In an era of heightened connectivity through digital platforms, students worldwide may be exposed to similar information, discourses, and awareness campaigns related to climate change. This shared global discourse could result in a uniform understanding and awareness of climate-related issues among students from different countries.

Another factor to consider is the possibility of common educational approaches influencing the outcomes of the study. Universities across the globe increasingly adopt international curricula and teaching methodologies. The integration of similar educational frameworks could contribute to a standardized level of knowledge and awareness about climate change among students, irrespective of their country of study. Furthermore, academic institutions often collaborate on research projects, fostering the exchange of knowledge and practices that may contribute to the observed uniformity in study results. Socio-cultural contexts could also play a pivotal role in shaping perceptions and responses to climate-related issues, thus contributing to the lack of differences among students from diverse regions. Shared cultural values, societal attitudes, and the prevalence of environmental concerns as part of a broader global consciousness may collectively influence how individuals, including students, perceive and respond to climate change [46].

In contrast to our study, research in Arab countries [17, 38] revealed divergent associations. Notably, Saudi students exhibited more positive attitudes toward environmental sustainability in healthcare compared to peers in Iraq, Egypt, and the Palestinian Territories, highlighting the complex interplay of geographical and educational factors in shaping attitudes. Moreover, the study uncovers disparities in knowledge levels, with Saudi Arabia and the Palestinian Territory students reporting significantly higher knowledge than their Egyptian and Iraqi counterparts. Most respondents, regardless of region, acknowledge the increasing health-related impacts of climate change, with over two-thirds anticipating further escalation in the next 20 years. This shared perception underscores a common awareness of the escalating threats posed by climate change to public health. These findings emphasize the need for a comprehensive examination of factors contributing to divergent attitudes and knowledge levels, enriching our understanding of the complex interplay between regional, educational, and societal influences on nursing students’ responses to climate change.

Moreover, another study delves into the attitudes of mental health nurses across different regions, further expanding our understanding of the complexities within this professional domain. While noting overarching similarities in the attitudes of these healthcare professionals, the research identifies nuanced differences that extend beyond the European context [47]. These disparities are suggested to be intricately linked to diverse cultural, societal, and organizational factors influencing mental health nursing practices. The study underscores the importance of recognizing and comprehending these variations to tailor effective interventions and training programs that consider the unique contextual influences on mental health nursing attitudes, ultimately contributing to enhanced patient care and well-being.

Furthermore, a study affirmed the link between psychological responses and climate-related attitudes, revealing that young climate activists experience higher levels of climate change anxiety compared to non-activists, with a nuanced finding indicating that greater knowledge among activists is associated with heightened feelings of hopelessness. This complex interplay underscores the intricate relationships between knowledge, activism, and psychological responses to environmental challenges [48]. Additionally, another study contributes significantly to understanding the behavioral impacts of global climate change, identifying connections between rising temperatures and adverse psychological outcomes. The study also explores specific behavioral outcomes tied to climate activism, revealing heightened climate change anxiety among activists and an amplification of hopelessness with increased knowledge [49]. Despite valuable insights, acknowledged limitations, such as potential biases in self-reported data and the need for further exploration of psychosocial processes, warrant consideration. Integrating findings from both studies advance our understanding of the multifaceted relationships between climate change, human behavior, and the varying influences of knowledge and activism on psychological well-being, refining our grasp of the behavioral ramifications of global climate change.

In connection with the broader discourse, other studies [50, 51] contribute a unifying conceptual framework to illuminate the intricate relationship between climate change and “within-country inequalities,” collectively referred to as “social inequality.” The study posits that this relationship operates within a vicious cycle, where initial inequality becomes a driving force, causing disadvantaged groups to bear a disproportionate burden of the adverse effects of climate change. This, in turn, leads to an exacerbation of subsequent inequality, creating a cyclical pattern of socio-environmental challenges. The available evidence highlighted in the study underscores the urgent need for comprehensive strategies that address both environmental sustainability and social justice to break this cycle and foster a more equitable and resilient society in the face of climate change.

Additionally, an international study conducted by Leal Filho et al., (2023) revealed differences in awareness and concern about climate change among university students across academic fields [52]. Business, administration, and law disciplines exhibited the lowest levels of both awareness and concern, while engineering, manufacturing, and construction fields demonstrated the highest levels. Biology and environmental sciences had the highest levels of awareness, while health and welfare disciplines expressed the highest levels of worry regarding climate change.

The results of the current study also showed that Jordanian students had significantly higher overall scores for attitudes toward climate change than students from other universities that had also been studied. They also showed significantly more positive attitudes toward climate change for nursing than students from other contributing universities. Given that the aforementioned Arab countries differ from one another, it may be possible to identify disparities in attitudes among students by comparing their customs, traditions, cultures, environments, way of life, and economic circumstances. Likewise, Richardson et al., (2016) observed notable variations among countries, with German nursing students demonstrating greater levels of sustainability awareness compared to their counterparts in the United Kingdom and Spain [53].

In a similar vein, Cruz et al., (2018) found that students from Saudi had more favorable attitudes about environmental sustainability in health care than students from Iraq, Egypt, and Palestine [54]. Attitudes toward environmental sustainability were significantly influenced by one’s place of residence, their community’s characteristics, and their knowledge of environmental issues and how they affect health from any nursing course. Moreover, Felicilda-Reynaldo et al., (2018) report that students’ attitudes regarding the environment fall into a spectrum, with major influences coming from variables connected to climate change, their residence country, their community kind, and their level of academic year [55].

The positive impact of academic achievement as the most significant predictor of psychological distress, as indicated by the strong positive beta coefficient (β = 0.381), may seem counterintuitive at first. However, it is consistent with previous research that has shown higher levels of academic pressure and stress among high-achieving students [56, 57]. One possible explanation for this unexpected finding is the phenomenon of academic pressure and its effects on psychological well-being. High-achieving nursing students often face heightened expectations from themselves, their peers, and faculty members. The pursuit of academic excellence in a rigorous nursing curriculum may lead to increased stress levels, anxiety, and psychological distress. These students may feel overwhelmed by the demanding coursework, clinical rotations, and the need to maintain high grades. Furthermore, perfectionistic tendencies commonly observed among high-achieving individuals could exacerbate feelings of inadequacy and self-doubt, contributing to psychological distress.

This suggests that nursing students who excel academically may experience increased psychological distress due to the demands and expectations placed upon them. The pressure to perform well academically can lead to a chronic state of stress, negatively impacting mental health and well-being. Moreover, the competitive nature of academic environments may foster feelings of comparison and self-evaluation, further intensifying psychological distress among high-achieving nursing students. Nursing education programs should be aware of this potential link between academic achievement and psychological distress and consider implementing support mechanisms to help students cope with stress and maintain their well-being. Providing resources such as counseling services, stress management workshops, peer support groups, and academic advising tailored to the unique needs of high-achieving students can mitigate the adverse effects of academic pressure on psychological health.

In the same spoken, the total score of knowledge regarding climate change significantly and positively correlated to the total score of attitude toward climate change. According to the views of numerous experts who have discussed the close relationship between knowledge and attitude, this relationship is virtually reasonable. All of this is to the student’s best advantage, as the more information develops, the better the attitude becomes, and vice versa. This supports the idea that increasing knowledge on a particular topic can lead to positive attitudes towards that topic [58]. This finding was supported by Ghazy and Fathy, (2023) who confirmed that there was a positive correlation between the total knowledge scores of nursing students with their total attitude scores regarding climate change [59].

The results also discovered a strong negative correlation between the overall psychological distress score and the overall knowledge and attitude score about climate change. Moreover, regression analysis presented the negative impacts of knowledge regarding climate change (β=-0.205) and attitude toward climate change (β=-0.045) on psychological distress align with research that highlights the potential benefits of climate change education and positive environmental attitudes. Previous studies have shown that individuals with greater knowledge about climate change and a positive attitude toward climate action tend to experience lower levels of distress and anxiety related to environmental issues [6]. This conclusion was strongly supported by Charlson et al., (2021) scoping review of the relationship between mental health and climate change, they found that exposure to climate-related conditions such as heat, humidity, rainfall, drought, wildfires, and floods has been linked to adverse effects on mental health [60]. These include psychological distress, increased mortality among individuals with pre-existing mental health conditions, higher rates of psychiatric hospitalizations, and an elevated risk of suicide.

It is important to note that while our study found significant associations, the percentage of variance explained (18.2%) suggests that other factors not included in the analysis may also contribute to students’ psychological distress. Future research could explore additional variables, such as social support, coping strategies, and personal resilience, to further enhance our understanding of the complexity of psychological distress among nursing students in the context of climate change.

### Limitations and strengths of the work

The current study has several limitations. Firstly, the use of a cross-sectional research design limits the ability to establish causal relationships. Therefore, future research employing longitudinal designs is recommended to assess the impact of students’ knowledge and attitudes toward climate change on their psychological distress over time. Additionally, the convenience sampling method may introduce selection bias, limiting the generalizability of our findings to similar populations in Middle Eastern contexts. While we acknowledge these limitations, it’s important to note the potential influence they may have had on the study findings and interpretations.

The trust in self-reported tools also poses a potential source of bias, as participants may provide socially desirable responses or inaccurately report their experiences. Furthermore, the assumption of a conservative response rate of 50% influenced our sampling strategy and calculation of the required sample size. However, the actual response rate significantly exceeded our expectations, reaching 96%. While this high response rate is advantageous for the robustness of our findings, our initial assumption of a lower response rate could be considered a limitation, potentially impacting the logistics and resource allocation for the study.

Moreover, the study focused solely on the relationship between psychological distress and students’ knowledge and attitudes regarding climate change, overlooking other potential predictors such as quality of life and psychological well-being. Future research should explore these factors to provide a more comprehensive understanding of the determinants of psychological distress among nursing students.

Despite these limitations, a notable strength of this study is the inclusion of a diverse sample from three different Middle Eastern countries. This diversity enhances the external validity of the findings and allows for greater generalizability to similar populations of nursing students in Middle Eastern contexts.

### Implications for nursing education and practice

Incorporating education about climate change and its potential health effects into nursing courses may be an effective strategy for reducing nursing students’ psychological distress and promoting positive attitudes towards environmental sustainability and climate action. Additionally, interventions aimed at improving academic achievement and reducing academic stress among nursing students may also have a positive impact on their psychological well-being. These findings have important educational implications for nursing programs.

First, results suggest that age may be an important factor to consider when designing educational interventions aimed at improving academic achievement. Interventions tailored to the specific developmental stages and needs of nursing students at different ages may be more effective in promoting academic success and mitigating psychological distress. For instance, younger nursing students may benefit from mentorship programs or peer tutoring, while older students may require targeted support in time management and study skills.

Second, they highlight the importance of including climate change education in nursing curricula to promote positive attitudes towards environmental sustainability and potentially reduce psychological distress related to climate change. Nursing programs can integrate climate change education into existing courses, such as public health, community nursing, and ethics. This integration can include modules on the health impacts of climate change, strategies for mitigating environmental risks in healthcare settings, and the role of nurses in advocating for environmental sustainability.

However, implementing these interventions may face challenges. One potential barrier is the limited availability of resources, including faculty expertise and curriculum development funds, to integrate climate change education into nursing curricula effectively. To overcome this challenge, nursing programs can collaborate with interdisciplinary teams, leverage external partnerships with environmental organizations or public health agencies, and seek grant funding to support curriculum development initiatives.

Another challenge is faculty resistance or lack of awareness about the importance of climate change education in nursing. Addressing faculty concerns and providing professional development opportunities on climate change and its health implications can help garner support for curriculum changes. Additionally, fostering a culture of environmental consciousness within nursing programs through faculty workshops, seminars, and awareness campaigns can create momentum for incorporating climate change education into the curriculum.

Furthermore, nursing programs should consider expanding interprofessional education opportunities to foster collaboration between nursing students and students from other healthcare disciplines, environmental sciences, and policy programs. Collaborative initiatives can enhance students’ understanding of the interconnectedness between climate change, health, and healthcare delivery, while also promoting teamwork skills essential for addressing complex environmental health challenges.

## Conclusion

In conclusion, this study has significantly enhanced our understanding of the intricate relationships among nursing students’ knowledge, attitudes toward climate change, and psychological distress. The findings reveal a positive correlation between knowledge and attitudes, indicating that a deeper understanding of climate change aligns with more favorable attitudes. Moreover, the study sheds light on the complex interplay involving psychological distress, academic achievement, and climate-related perceptions. Interestingly, while academic achievement shows a positive correlation with psychological distress, knowledge and a positive attitude towards climate change demonstrate a mitigating effect on psychological distress. These nuanced correlations highlight the importance of fostering a comprehensive understanding of climate change among nursing students, coupled with a positive attitude, as potential strategies for reducing psychological distress.

In light of these insights, recommendations for educational interventions and support mechanisms emerge. To address the observed positive correlation between academic achievement and psychological distress, educational institutions may consider integrating stress management programs and mental health support into their academic curriculum. These programs could include workshops on stress reduction techniques, counseling services, and peer support groups aimed at providing students with coping mechanisms and resources to manage academic pressures effectively.

Additionally, promoting climate change education tailored to nursing students can serve a dual purpose: enhancing knowledge and cultivating positive attitudes. Integrating climate change education into nursing curricula, as suggested earlier, can provide students with the necessary knowledge and skills to understand the health impacts of climate change and the role of nurses in addressing environmental challenges. Furthermore, fostering positive attitudes towards environmental sustainability through experiential learning opportunities, such as field trips to sustainable healthcare facilities or engagement in community-based environmental initiatives, can instill a sense of responsibility and agency among nursing students.

Such interventions may not only contribute to lowering psychological distress but also foster a generation of healthcare professionals with an increased awareness of climate-related challenges and a positive mindset towards addressing them. By equipping nursing students with the knowledge, skills, and attitudes necessary to confront the health impacts of climate change, educational institutions can empower future nurses to advocate for environmentally sustainable healthcare practices and contribute to broader efforts aimed at mitigating climate change effects on health. Ultimately, these recommendations aim to create a more resilient and informed nursing workforce capable of navigating the complex intersections of climate change, mental health, and academic success.

### Electronic supplementary material

Below is the link to the electronic supplementary material.

Supplementary Material 1

## Data Availability

The datasets generated during and analyzed during the current study are not publicly available due to confidentiality agreements but are available upon reasonable request from the corresponding author.
